# Combined homocysteine and apoE rs429358 and rs7412 polymorphism in association with serum lipid levels and cognition in Chinese community-dwelling older adults

**DOI:** 10.1186/s12888-022-03877-4

**Published:** 2022-03-29

**Authors:** Chunxiu Wang, Xunming Ji, Zhe Tang, Zhongying Zhang, Xiang Gu, Xianghua Fang

**Affiliations:** 1 Department of Evidence-based Medicine, Xuanwu Hospital, Capital Medical University, No 45 Changchun Street, Xicheng District, Beijing, China; 2 Department of Neurological Surgery, Xuanwu Hospital, Capital Medical University, Beijing, China; 3 Geriatric Department, Xuanwu Hospital, Capital Medical University, Beijing, China; 4 Geriatric Department, Youyi Hospital, Capital Medical University, Beijing, China

**Keywords:** Apolipoprotein E, Polymorphism, Total homocysteine (tHcy), The mini-mental scale examination (MMSE) score, Cognitive function, Aging

## Abstract

**Background:**

ApoE gene polymorphism and serum total homocysteine (tHcy) has been reportedly associated with cognition. In this study, we assessed the association of combined ApoE gene polymorphism and tHcy with cognition in Chinese elder adults.

**Methods:**

A cross- sectional study was carried out by recruiting 1458 community-dwelling people aged 55+ and above in Beijing in 2009. All participants were interviewed using a standard questionnaire and underwent a physical examination. The mini-mental scale examination (MMSE) score was used in assessing cognitive function. Fasting venous blood samples were taken for ApoE rs429358, rs7412 genotyping, tHcy and other serum lipid measurements.

**Results:**

Participants with high serum tHcy level showed a relatively lower orientation, attention abilities as well as the total MMSE score than the group with normal tHcy after adjusting confounding factors. ApoE rs429358 and rs7412 variants were observed to have the highest serum TC and TG level in the subjects with high serum tHcy level (*p* <  0.05). Cognition of the subjects was found to be significantly associated with high serum tHcy level and ApoE genetic polymorphism (*p* <  0.05). Independent of age, BMI, education levels, smoking and alcohol drinking, the worst cognitive ability were detected in the high serum tHcy level subjects with ApoE rs429358C/T and rs7412 C/T as compare with other groups, especially orientation function, memory and delayed recall ability and attention ability.

**Conclusion:**

High serum tHcy level in combination with ApoE rs429358 and rs7412 variants might be linked with serum lipid levels and cognition, particularly for orientation function and memory and delayed recall ability in old Chinese adults.

**Supplementary Information:**

The online version contains supplementary material available at 10.1186/s12888-022-03877-4.

## Introduction

Homocysteine is a nonessential amino acid derived from metabolism of dietary-obtained methionine through demethylation. There is clear evidence that elevated serum total homocysteine (tHcy) is a modifiable risk factor for development of vascular diseases, cognitive decline, dementia, as well as other psychogeriatric conditions [1]. In particular, high serum tHcy level has been implicated in poorer performance in numerous cognitive domains, including attention, executive function, recall memory and overall cognitive functions [2]. However, the existing studies have reported inconsistent associations between high serum tHcy level and different cognitive domains. Some previous studies have revealed that high serum tHcy level is associated with poorer executive-language functioning but not with memory in a non-demented elderly population, and also found in subjects with mild Alzheimer’s disease and vascular dementia, when compared with cognitively normal subjects [3]. Some researches showed that B vitamin treatment slowed the rate of decline in the Mini-Mental State Examination (MMSE) over the 18-month period of the trial [4, 5], however, it has already been well documented that homocysteine-lowering with B vitamin supplements does not appear to improve cognitive function in individuals with or without existing cognitive impairment in several meta-analyses of RCTs [6–8]. Polymorphism for the apolipoprotein (ApoE) gene may also increase the effect of tHcy on cognitive decline.

ApoE is known to be lipid-binding proteins involved in the transport of lipids in plasma. Several studies suggested that ApoE has a major physiological role in the regulation of overall lipid and lipoprotein homeostasis. Prospective studies have shown that the ApoE polymorphism has a substantial influence on the metabolism of blood lipids in different populations [9]. In addition, a recent comprehensive meta-analysis demonstrated carriers of the ApoE gene variants have higher total lipoprotein and low-density lipoprotein cholesterol levels than noncarriers [10]. Moreover, ApoE polymorphism, particularly the ApoE-ε4, which derives from rs429358 and rs7412 polymorphism, represents a genetic predisposing factor for developing AD and cognitive impairment [11]. Consequently, there is considerable interest in understanding whether ApoE polymorphism influences cognition in AD and non-demented subjects as well as healthy subjects. Despite a substantial literature reporting effects of the presence of ApoE polymorphism on cognition of healthy older people, findings are less consistent [12], with some studies showing no existing relationship and others showing a worse cognitive performance in ApoE variants [13]. However, the results of these meta-analyses suggest that ApoE polymorphism does affect cognitive performance in healthy aging adults; the impact is relatively small and may relate to specific cognitive ability domains, especially on measures of episodic memory, executive functioning, and overall global cognitive ability [14, 15]. Notably, despite the evidence that the ApoE polymorphism may modify the effects of tHcy on serum lipid level and cognition, only a few studies have testified the hypothesis that ApoE -ε4 allele modifies the associations between plasma tHcy and cognitive performance in the elderly, with both negative [16, 17] and positive effects [2, 18]. Some authors have suggested that high tHcy level, when combined with ApoE variants might be transfer a greater risk primarily in executive dysfunctions rather than memory loss [4].

While previous studies have reported significant cognitive impairment in ApoE variants carriers compared to non-carriers across a range of cognitive domains, but the results of studies conducted in all these areas have been inconclusive, which were likely to be related to ethnic difference. Moreover, few studies have testified that the ApoE gene polymorphism modifies relations between serum tHcy and different cognitive domains in older Chinese adults, which deserves further investigation. In this study, we used baseline data from a community-based dwellers to examine the influence of tHcy and ApoE polymorphism (e.g., ApoE rs429358 and rs7412) on serum lipid level and cognition, and also their combined effects on serum lipid level and cognition in old Chinese adults.

## Methods

### Study population

This was a secondary analysis of the Beijing Longitudinal Study of Aging (BLSA), a representative cohort study of community-dwelling Chinese people aged 55 years and over (response rate 91.2%). The study was initiated in 1992 and finished in 2015. The details of BLSA design, setting, sampling technique, questionnaires, physical examination, blood sample collection, and laboratory measurement were described elsewhere [19, 20]. The present analysis was based on the 2009 cohort, in which a total of 1458 participants without a history of stroke or MI completed a baseline health interview and a physical examination.

### Data collection

Information on demographic characteristics (sex, age), lifestyle factors [e.g., smoking (yes or no), alcohol drinking (yes or no)] were collected using a standardized questionnaire based on face-to-face interviews by trained interviewers. Body mass index was derived from the measurements as weight (kilogram) divided by the square of height (meter). Data on smoking and drinking status were collected by self-reporting. Educational level was classified into three categories (illiterate, primary school, high school and above). Physical activities was classified by the number of times in a week, < 2 times weekly and ≥ 2 times weekly. Individual food consumption data were collected through one consecutive 24-h recalls combined with a food inventory.

### Cognitive test

Cognitive function was assessed by Mini-Mental state Examination (MMSE), a commonly used screening measure of cognitive impairment in general practice, which consists of five cognitive domains including orientation function, memory and delayed recall, attention, language, visual and executive ability. This assessment was validated and has been widely used in other large-scale or cross-sectional studies on cognitive function in the elderly previously [21, 22]. The test was carried out by trained neurologists in Xuanwu Hospital.

### Laboratory measurements

For biochemical analysis, all fasting blood samples were drawn in the morning, and centrifuged at 3000 rpm for 15 min at 4 °C, and then transferred to a central laboratory (IPE Center for Clinical Laboratory, Beijing, China), which performed all analyses within 24 h. A Hitachi 7600 clinical chemistry analyzer (Hitachi, Tokyo, Japan) was used to determine serum total cholesterol (TC) and triglyceride (TG). Fasting glucose was determined by the glucose oxidase–peroxidase method. High density lipoprotein cholesterol (HDL-C) and low-density lipoprotein cholesterol (LDL-C) were measured by the direct assay method. Homocysteine concentration was measured by enzymatic cycling assay (Architect-i 2000; Abbott, Texas, America) and considered to be the sum of the homocysteine, and the inter-assay coefficients of variation (CV) were less than7.0%. We defined serum tHcy < 15 μmol/l and ≥ 15 μmol/l as absence or presence of hyperhomocysteinemia, which was consistent with the criteria used in previous studies [23].

Peripheral blood samples (5 ml) were collected in vacuum tubes and stored at − 80 °C. Genomic DNA was extracted from frozen peripheral blood using the Wizare genomic.

DNA purification kit (Promega, Madison, WI, USA). DNA concentration and purity were accessed at 260/280 absorbance by a NanoDrop spectrophotometer. The DNA extraction was stored at − 20 °C until genotyping. Genotyping for ApoE rs429358 and rs7412 was performed on a MassArray system (Sequenom iPLEX assay, BGI Tech, Beijing, China), which is based on a multiplex PCR reaction, a locus-specific single-base extension reaction, and matrix-assisted laser desorption ionization time-of-flight mass spectrometry.

### Statistical analysis

Sample characteristics were described using means and standard deviations or mean (95% confidence interval, CI) for continuous data and percentages for categorical data; Participants were classified according to two groups of tHcy and categories of ApoE rs429358 and rs7412 genotypes. The GLM univariate analysis was used to compare the means of the detected parameters between the groups. Interaction between each variant and homocysteine level was examined, after adjusted some potential confounding factors including age, sex, BMI, education, smoking, alcohol drinking, physical activity. For comparing differences in TG, TC, LDL-C, HDL-C, confounding factors including age, sex, BMI, smoking, alcohol drinking, physical activity were adjusted; When comparing homocysteine level or ApoE genotype differences in cognition (MMSE score), confounding factors including age, sex, BMI, education, smoking, alcohol drinking, and physical activity were adjusted. Multiple linear regression models were developed to examine the combined effects of tHcy and ApoE genotype on blood lipid level and cognitive function, controlling for age, sex, education, BMI, smoking, alcohol drinking, physical activity. The level of significance was set as *p* < 0.05 (2-tailed). Data analysis was performed using SPSS version 22.0.

## Results

### Characteristics of the study population

Table 1 shows the baseline characteristics of participants. The 1458 participants (652 males and 806 females) had an average age of 69.5 ± 8.1 years. The mean (standard deviation [SD]) tHcy was 21.3(18.2) mmol/L. Participants with higher serum tHcy level were slightly older, 70.5 (8.2) versus 67.7 (7.7). Compared to participants with normal tHcy, those with high serum tHcy level were more likely to be male, less-well educated, and to smoke cigarettes. Participants of the latter group were also more likely to have higher systolic blood pressure (SBP), pulse pressure (PP), uric acid (UA) level, higher scores of activities of daily living (ADL) and instrumental activities daily living (IADL) (*p* < 0.05). However, no significant differences were found across other risk factors (e.g., BMI, DBP, TC, TG, LDL-C, HDL-C, Fasting blood sugar, GDS score) between the two groups (Tables 1 and 2), also there is no correlation between the genotype frequencies of ApoE rs429358 and ApoE rs7412 and tHcy levels.

**Table 1:** Baseline characteristics of the sample as grouped by tHcy

	Total	HCY ≤ 15	HCY > 15	t/X^2^	*p* value
N	1458	556	902		
Age (yrs)	69.5 ± 8.1	67.7 ± 7.7	70.5 ± 8.2	6.66	< 0.001*
Female,n (%)	806 (55.3)	374 (67.3)	432 (47.9)	52.22	< 0.001*
Education,n (%)				30.06	< 0.001*
illiterate,n (%)	439 (30.1)	128 (23.0)	311 (34.5)		
primary school,n (%)	485 (33.3)	181 (32.6)	304 (33.7)		
High school,n (%)	534 (36.6)	247 (44.4)	287 (31.8)		
BMI(kg/m^2^)	24.2 ± 4.1	24.3 ± 3.7	24.2 ± 4.3	0.88	0.378
Smoking,n (%)	458 (31.5)	124 (22.3)	334 (37.1)	34.55	< 0.001*
Drinking,n (%)	437 (30.0)	133 (23.9)	304 (33.8)	16.01	< 0.001*
SBP, mmHg	139.5 ± 20.4	137.2 ± 20.1	140.9 ± 20.5	3.30	< 0.001*
DBP, mmHg	76.7 ± 11.0	76.8 ± 11.3	76.7 ± 10.9	0.21	0.837
PP, mmHg	62.8 ± 17.4	60.4 ± 16.6	64.2 ± 17.8	4.01	< 0.001*
Fasting blood sugar, mmol/L	5.8 ± 1.7	5.9 ± 1.9	5.7 ± 1.7	2.95	0.081
UA, mmol/L	351.5 ± 96.8	331.1 ± 84.1	364.0 ± 101.9	6.38	< 0.001*
GDS score	5.7 ± 5.7	5.9 ± 5.0	5.6 ± 4.9	2.51	0.114
ADL score	6.2 ± 1.1	6.1 ± 0.6	6.2 ± 1.4	18.03	< 0.001*
IADL score	6.7 ± 2.2	6.4 ± 1.7	6.8 ± 2.3	48.42	< 0.001*
**Genotype**					
ApoE, rs429358				0.42	0.520
C/T, n (%)	184 (14.8)	74 (15.6)	110 (14.2)		
T/T, n (%)	1063 (85.2)	401 (84.4)	662 (85.8)		
ApoE, rs7412				0.24	0.624
C/C, n (%)	1020 (82.7)	390 (83.3)	630 (82.2)		
C/T, n (%)	214 (17.3)	78 (16.7)	136 (17.8)		

Data are presented as mean (standard deviation), otherwise as indicated

*SBP* Systolic Blood Pressure, *DBP* Diastolic Blood Pressure, *PP* Pulse Pressure, *BMI* Body Mass Index, *UA* Uric Acid, *ADL* Activities of Daily Living, *ApoE* Apolipoprotein E. *p* < 0.05 was considered as significance

**Table 2:** Subject serum parameters according to tHcy in Chinese adults

	Total	HCY ≤ 15	HCY > 15	t/X^2^	*p* value
N	1458	556	902		
TC, mmol/L	5.9 ± 1.1	5.9 ± 1.2	5.8 ± 1.1	1.09	0.297
TG, mmol/L	1.6 ± 1.1	1.7 ± 1.3	1.6 ± 1.1	3.04	0.080
LDL-C, mmol/L	3.0 ± 0.7	3.0 ± 0.8	2.9 ± 0.7	1.62	0.203
HDL-C, mmol/L	1.2 ± 0.3	1.2 ± 0.3	1.2 ± 0.3	0.54	0.461
HCY, mmol/L	21.3 ± 18.2	11.9 ± 2.1	27.2 ± 21.0	7.08	< 0.001*

Data are presented as mean (standard deviation), otherwise as indicated

*TC* Total Cholesterol, *TG* Triglyceride, *HDL-C* High-density Lipoprotein Cholesterol, *LDL-C* Low-density Lipoprotein Cholesterol, *HCY* Homocysteine. *p* < 0.05 was considered as significance

### Cognition according to presence or absence of high serum tHcy level

The difference of cognitive function was observed in the groups with absence or presence of high serum tHcy level, after adjusting age, BMI, education levels, smoking, alcohol drinking, as shown in Table 3. Participants with high serum tHcy level demonstrated a relatively lower orientation, attention abilities as well as the total MMSE score than the group with normal tHcy (*p* < 0.05). No statistical significance was detected on other cognitive domains between the two groups (*p* > 0.05).

**Table 3:** Cognition according to tHcy in Chinese adults

	Total	HCY ≤ 15	HCY > 15	*F*	*p* value
N	1458	556	902		
Orientation	8.59(8.47,8.71)	8.88(8.83,9.10)	8.24(8.13,8.41)	4.32	0.032*
Memory and delayed recall	5.26(5.20,5.33)	5.32(5.23,5.41)	5.26(5.18,5.36)	1.60	0.206
Attention	3.94(3.83,4.05)	4.24(4.08,4.40)	3.83(3.59,4.01)	9.80	0.002*
Language	6.45(6.38,6.52)	6.51(6.22,6.71)	6.44(6.16,6.64)	1.23	0.268
Visual and executive	0.66(0.63,0.68)	0.73(0.69,0.76)	0.62(0.59,0.73)	1.16	0.282
MMSE score	24.90(24.58,25.22)	25.50(25.26,25.83)	24.60(24.27,25.03)	6.06	0.014*

Data are presented as mean (95%CI). *MMSE* Mini-Mental State Examination

The General Line Model (GLM) univariate analysis was used for data analysis. Factors including sex, age, education, BMI, smoking, alcohol drinking and physical activity were adjusted. *p < 0.05 was considered as significance

### Subject serum parameters according to ApoE polymorphism

The ApoE genotype difference of subject characteristics and serum parameters was presented (Supplementary Table 1). Comparing the subjects with ApoE rs7412 C/T genotype, subjects with ApoE rs7412 C/C genotype have lower serum TC, TG levels and higher LDC-C level (*p* < 0.05). However, ApoE rs429358 polymorphism has no effect on TC, TG, HDL-C, LDL-C (*p* > 0.05).

### Subject serum parameters according to ApoE polymorphism and presence or absence of high serum tHcy level

As can be seen in Table 4, we found significant combined effects of tHcy and ApoE rs429358 variant on serum parameters. The subjects with high serum tHcy level and ApoE rs429358 C/T genotype have the highest serum TC, TG levels compared with other groups (*p* < 0.05). However, the interaction between ApoE rs429358 and tHcy level on the serum TC and TG level was not detected (p_interaction_ = 0.678; p_interaction_ = 0.344).

**Table 4:** Subject serum parameters according to tHcy and ApoE rs429358 in Chinese adults

	HCY ≤ 15		HCY > 15		*p* value
	ApoE rs429358(C/T)	ApoE rs429358(T/T)	ApoE rs429358(C/T)	ApoE rs429358(T/T)	
N	74	401	110	662	
TC, mmol/L	5.70(5.25,6.14)	5.83(5.65,6.01)	6.19(5.69,6.59)	5.81(5.68,5.95)	0.021*
TG, mmol/L	1.45(1.01,1.89)	1.63(1.46,1.81)	1.87(1.42,2.21)	1.55(1.42,1.68)	0.030*
LDL-C, mmol/L	3.01(2.71,3.30)	2.99(2.87,3.11)	3.02(1.15,1.33)	2.88(2.79,2.98)	0.407
HDL-C, mmol/L	1.20(1.10,1.31)	1.22(1.18,1.27)	1.24(1.12,1.25)	1.22(1.19,1.25)	0.482
HCY, mmol/L	12.47(5.61,19.32)	12.22(9.46,14.96)	24.40(18.25,30.55)	27.77(25.74,29.80`)	0.000*

Data were expressed as mean (95% CI).The General Line Model (GLM) univariate analysis was used for data analysis. Factors including sex, age, BMI, smoking, alcohol drinking and physical activity wereadjusted. *TC* Total Cholesterol, *TG* Triglyceride, *HDL-C* High-density Lipoprotein Cholesterol, *LDL-C* Low-density Lipoprotein Cholesterol, *HCY* Homocysteine, *ApoE* Apolipoprotein E

As presented in Table 5, the statistical analysis showed the highest serum TC and TG level in individuals with high serum tHcy level and ApoE rs7412 C/T genotype (*p* < 0.05). Whereas, the serum LDL-C levels significantly lowered in both ApoE rs7412 C/T genotype groups (*p* < 0.001), especially the group with high serum tHcy level. The interaction of ApoE rs7412 and serum tHcy level on the serum LDL-C level was observed (p_interaction_ < 0.001).

**Table 5:** Subject serum parameters according to tHcy and ApoE rs7412 in Chinese adults

	HCY ≤ 15		HCY > 15		*p* value
	ApoE rs7412(C/C)	ApoE rs7412(C/T)	ApoE rs7412(C/C)	ApoE rs7412(C/T)	
N	390	78	630	136	
TC, mmol/L	5.48(5.21,5.74)	5.67(5.53,5.97)	5.53(5.30,5.75)	5.85(5.75,5.96)	0.006*
TG, mmol/L	1.50(1.44,1.66)	1.57(1.39,1.69)	1.63(1.40,1.85)	1.89(1.63,2.16)	0.024*
LDL-C, mmol/L	3.04(2.96,3.12)	2.62(2.43, 2.80)	2.95(2.88,3.02)	2.33(2.06,2.61)	< 0.001*
HDL-C, mmol/L	1.22(1.18,1.27)	1.21(1.18,1.25)	1.21(1.18,1.24)	1.28(1.19,1.34)	0.225
HCY, mmol/L	12.41(11.00,13.82)	12.46(9.36,15.55)	28.03(25.69,30.38)	26.47(22.26,30.68)	< 0.001*

Data were expressed as mean (95% CI). The General Line Model (GLM) univariate analysis was used for data analysis. Factors including sex, age, BMI, smoking, alcohol drinking and physical activity were adjusted. *TC* Total Cholesterol, *TG* Triglyceride, *HDL-C* High-density Lipoprotein Cholesterol, *LDL-C* Low-density Lipoprotein Cholesterol, *HCY* Homocysteine, *ApoE* Apolipoprotein E

### ApoE genotype differences with cognition

No relationship between ApoE rs429358 polymorphism and cognitive function was detected in old Chinese adults (*p* > 0.05). The subjects with ApoE rs7412 C/T significantly affected orientation function (*p* < 0.05), and there were no significant effects of ApoE genotype on other cognitive domains as well as total MMSE score in old Chinese adults (*p* > 0.05) **(**Supplementary Table 2).

### The presence or absence of high serum tHcy level and ApoE genotype differences with cognition

The significant combine effect of ApoE genotype and tHcy on cognitive function was presented in Tables 6 and 7. Comparing with the subjects with other genotypes, the subjects with ApoE rs429358 C/T and with normal tHcy have the highest orientation and memory and delayed recall ability, and the lowest orientation and memory and delayed recall ability were observed in the high serum tHcy level subjects with ApoE rs429358 C/T (*p* < 0.05). As shown in Table 7, Carriers of ApoE rs7412 C/T with normal tHcy have the highest orientation and attention ability as well as highest total MMSE score comparing with subjects with other genotypes (*p* < 0.05). The interaction of ApoE rs7412 and serum tHcy level on the total MMSE score was observed (p_interaction_ < 0.001). No difference were found on other cognitive domains between these four groups among old Chinese adults (*p* > 0.05).

**Table 6:** Cognition according to tHcy and ApoE rs429358 in Chinese adults

	HCY ≤ 15		HCY > 15		*p* value
	ApoE rs429358(C/T)	ApoE rs429358(T/T)	ApoE rs429358(C/T)	ApoE rs429358(T/T)	
N	74	401	110	662	
Orientation	8.80 (7.83,9.37)	8.50 (8.30,9.18)	8.37 (7.73,9.00)	8.60 (8.32,8.91)	0.039*
Memory and delayed recall	5.34 (5.04,5.59)	5.28 (5.08,5.53)	5.00 (4.48,5.10)	5.30 (5.23,5.45)	0.045*
Attention	4.38 (3.97,4.80)	4.16 (3.97,4.35)	3.69 (3.09,3.96)	3.91 (3.75,4.08)	0.378
Language	6.61 (6.32,6.89)	6.63 (6.50,6.75)	6.14 (5.75,6.33)	6.52 (6.41,6.63)	0.763
Visual and executive	0.62 (0.52,0.73)	0.71 (0.66,0.76)	0.58 (0.50,0.74)	0.60 (0.55,0.70)	0.210
MMSE score	25.52 (24.12,27.05)	25.13 (24.24,26.39)	24.13 (22.57,25.00)	24.98 (24.41,25.61)	0.640

*MMSE* Mini-Mental State Examination, *ApoE* Apolipoprotein E. The General Line Model (GLM) univariate analysis was used for data analysis. Factors including age, sex, BMI, education, smoking, alcohol drinking and physical activity were adjusted.*p < 0.05 was considered as significance

**Table 7:** Cognition according to tHcy and ApoE rs7412 in Chinese adults

	HCY ≤ 15		HCY > 15		*p* value
	ApoE rs7412(C/C)	ApoE rs7412(C/T)	ApoE rs7412(C/C)	ApoE rs7412(C/T)	
N	390	78	630	136	
Orientation	8.92 (8.69,9.14)	9.14 (8.65, 9.62)	8.68 (8.48,8.88)	8.22 (7.80,8.65)	0.048*
Memory and delayed recall	5.40 (5.27,5.53)	5.38 (5.11,5.66)	5.23 (5.12,5.35)	5.50 (5.26,5.74)	0.170
Attention	4.16 (3.97,4.35)	4.37 (3.96,4.78)	3.90 (3.73,4.07)	3.62 (3.26,3.98)	0.014*
Language	6.60 (6.47,6.73)	6.77 (6.48,7.05)	6.44 (6.33,6.56)	6.55 (6.30,6.79)	0.153
Visual and executive	0.71 (0.66,0.75)	0.65 (0.54,0.75)	0.66 (0.62,0.70)	0.65 (0.56,0.75)	0.380
MMSE score	25.78 (25.24,26.32)	26.30 (25.13,27.47)	24.92 (24.43,25.40)	24.55 (23.52,25.57)	0.041*

*MMSE* Mini-Mental State Examination, *ApoE* Apolipoprotein E. The General Line Model (GLM) univariate analysis was used for data analysis. Factors including age, sex, BMI, education, smoking, alcohol drinking and physical activity were adjusted. *p < 0.05 was considered as significance

Meanwhile, multiple linear regression models were developed to examine the combined effects of tHcy and ApoE genotype on blood lipid level and cognitive function, introducing the serum tHcy level and ApoE genotype interaction terms, explore the combined effect of the serum tHcy level and ApoE rs7412 on the serum LDL-C level (β = − 0.138, *p* < 0.001), the orientation ability (β = − 0.273, *p* < 0.001) and attention ability (β = − 0.186, *p* = 0.001) and total MMSE score (β = − 0.584, *p* = 0.001). However, the interaction of ApoE rs429358 genotype and the serum tHcy level was not associated with the serum LDL-C level (β = − 0.021, *p* = 0.147) and total MMSE score ((β = − 0.584, *p* = 0.254)), when considered independently of the covariates.

### Dietary intakes according to tHcy

In contrast to individuals with high serum tHcy level, subjects with normal tHcy reported to have a higher intake of red meat, poultry, fish, eggs, milk, bean, and fruit, whereas subjects with high serum tHcy level had a higher intake of staple food and salty taste preference (*p* < 0.05) (Supplementary Table 3).

## Discussion

In this cross-sectional study, we present data that describe significant differences of sex, age, educational level, lifestyle, dietary intakes, SBP, PP, UA, ADL and IADL between normal tHcy and high serum tHcy level group. Meanwhile, a significant negative correlation between tHcy and MMSE scores were observed in aging Chinese population. Also, here, in adjusted analysis by employing The GLM univariate analysis, the statistically significant associations were obtained between genetic variation in ApoE rs7412 and serum TC, TG and LDC-C levels, whether stratified by tHcy or not, the results are the same, in other word, the subjects with high serum tHcy level and rs7412 C/T genotype have highest TC, TG and lowest LDL-C level than the subjects with C/C genotype. This finding shows consistency with the results from previously studies [24, 25], also partial consistent with some published reports [26, 27]. In the present study, we did not observe the relationship between ApoE rs429358 and serum TC, TG, Glu and LDC-C levels, our findings are consistent with the results of similar studies performed in old Chinese populations [28, 29]. Meanwhile, it’s worth noting that the subjects with high serum tHcy level and ApoE rs429358 C/T have the highest serum TC and TG levels after including tHcy level into the data analysis, these results indicated that the addition of high serum tHcy level will increase the serum TC and TG levels in the subjects with ApoE rs429358 C/T genotype compared to subjects with ApoE rs429358 T/T. However, we did not detect the interaction between ApoE rs429358 and tHcy level on the serum TC and TG level (p_interaction_ = 0.678; p_interaction_ = 0.344).

Furthermore, we also found the variant of ApoE rs7412 was associated with orientation ability, and no effect of ApoE rs7412 and rs429358 polymorphism on other cognitive function was observed in the elderly, which was in accordance with the Brazil Cohort Study and the southern Italian study [30, 31]. In their research, the authors reported that ApoE rs7412 and rs429358 polymorphism have no significant effects on cognitive performance that can be detected even in the MMSE test. In addition, they did observe that ApoE variation was not correlated to the overall cognitive performance as evaluated by the MMSE screening test. In our study, as expected, we also observed elevated serum tHcy level associated specifically with lower orientation, attention abilities and total MMSE scores in aging Chinese population, particularly, after adjustment for other covariates, the combined effects of ApoE rs429358 and high serum tHcy level on the orientation ability and memory and delayed recall ability was detected. We also found the influence of ApoE rs7412 and high serum tHcy level on orientation and attention ability as well as total MMSE score. We also observed the interaction of ApoE rs7412 and serum tHcy level on the total MMSE score in the cross-sectional study. These results indicated that ApoE rs7412 polymorphism could modify the influence of high serum tHcy level on cognitive function. However, future research is needed to further clarify the influence of ApoE polymorphism and serum tHcy level on cognitive impairment with the larger and longitudinal study.

As described before, serum level of tHcy are influenced by dietary factors. In our study, we observed a significant difference in dietary intake between subjects with normal tHcy and high serum tHcy level. Subjects with normal tHcy consumed more high protein and low fat foods and more fruits as well as less staple and salty food than the subjects with high serum tHcy level. However, there is no significant difference in blood lipid level between these two groups. These results indicate that consumption of foods containing fat does not necessarily reflect the lipid metabolism in elderly adults. Moreover, our findings are in agreement with previous investigations proposing that the unfavourable effects of ApoE rs7412 and rs429358 polymorphism on the lipid profile in subjects with high serum tHcy level [29, 32], which suggest that some special environmental or dietary conditions may lead to the potential disadvantage of the ApoE genotype. Therefore, further studies are needed to verify the complex interaction of the ApoE polymorphism with environmental and dietary factors and its impact on serum lipid and cognition.

However, the relationship of ApoE gene polymorphism with serum lipid metabolism and cognition is still controversial between our study and other studies might be due to different research methods (e.g., cross-sectional or cohort studies) and discrepancy in study population characteristics, such as age, sex, race, as well as statistics (e.g., small sample sizes, the lack of statistical power, different statistical analysis methods). Particularly, findings on the lacking of consistency in these relationships among different ethnic populations have been.

Reported [33]. Nevertheless, consistently with our findings, an increased degree of correlation of tHcy and cognitive performance has been noted in a cohort of carriers of ApoE variants [18].

Both in vitro and in vivo experiments demonstrated that tHcy induces neuronal damage and cell loss by both excitotoxicity and different apoptotic processes [34]. Clinical evidence suggested elevated tHcy level contributes to neuronal degeneration in age-related or stress-related neuropsychiatric disorders in healthy elderly subjects as well as in elderly at risk of Alzheimer’s disease [17, 35]. On the other hand, substantial literature has reported that the ApoE gene was characterized for its impact on lipids metabolism, neuronal function, as well as in the development of certain pathogenesis of neurological diseases [36, 37]. In addition, a recent review suggested that two compatible neurobiological mechanisms (prodromal and phenotype) by which the ApoE genotype influences cognition possibly lead to the different cognitive consequences of neurobiological processes in healthy adults [38].

To date, the connection between ApoE and tHcy has been demonstrated by some previous studies; however, the molecular mechanism that underlies the specific effects of ApoE on the tHcy level has not been completely established. Additionally, few studies have reported ApoE genotype is associated with Neuron damage and repair [39], the combined effect of tHcy and ApoE polymorphism might be more than an accumulation of different detrimental effects, instead, ApoE genotype has been implicated in neuronal susceptibility to impairment. Another explanation is that tHcy affects the development of cognitive impairment in various pathways, which all may be modulated by the ApoE polymorphism. In particular, it is important to note that, a recent study did show that the validity of the predictive model for an association of the ApoE polymorphism with AD was increased when tHcy levels were added to the tested regression [40].

Our results suggest that ApoE rs429358 variant reveal unsusceptible to cognitive decline, while ApoE rs7412 variant imply its unfavourable influence on cognitive function in these older Chinese adults, independently of tHcy levels. However, when ApoE variants (e.g., rs429358C/T or rs7412 C/T) are combined with the presence of the high tHcy it is a particularly unfavourable circumstance. High tHcy can be prevented with the replacement of folic acid and B-vitamins, and early preventive intervention may be required to further illuminate whether cognitive function improves after adequate B vitamins and folic acid intake by supplementation to normalize tHcy levels. Although the conclusions of these associations remain discrepant, identification the roles of these gene variants is therefore of critical importance to further exploring an attenuating effect of tHcy lowering therapy.

Our data must be interpreted with caution. Firstly, the study used the cross-sectional detection of the association between tHcy and ApoE polymorphism and cognition, which limits the interpretation of cause and effect; also it is important to reproduce the analysis in further prospective studies. Secondly, cognitive screening instruments such as the MMSE do not cover all key cognitive domains and also lack sensitivity to detect subtle and early signs of cognitive impairment [41]. Although a serial of specific tests with well-validated psychometric properties are widely accepted in clinical practice, they are not applicable to community-based epidemiological studies due to its complex assessments. To date, global cognitive tests remain the most applicable tools for quick screen of cognitive functions in these studies, especially when environmental conditions may interfere with participants’ attention [7, 42]. Thirdly, the genotype frequencies of ApoE gene in different racial and ethnic groups show different patterns. Besides, the relationship between ApoE polymorphism and tHcy with blood lipid parameters might vary depending on different genetic groups and the nature geographical factors including diverse lifestyles and eating habits. Therefore, caution must be taken in extrapolating this relationship to others. Additionally, the sample size was relatively small for this analysis when it was stratified by ApoE genotype status. The further studies with a large cohort are encouraged to provide scientific evidence of the role of tHcy and ApoE polymorphism in cognition in representative elderly.

## Conclusion

In conclusion, our results suggest that ApoE polymorphism and tHcy might play potential roles in serum lipid level and cognition function in aging Chinese adults. The elder adults with high tHcy and ApoE rs429358 C/T have the highest serum TC and TG levels. High tHcy in combination with gene polymorphism (e.g., rs429358C/T or rs7412 C/T) might be associated primarily with orientation function and memory and delayed recall ability. The tHcy level is liable to develop impaired cognitive function, particularly among elder adults with ApoE gene polymorphism.

## Supplementary Information

**Additional file 1.**

## Data Availability

The datasets used and/or analyzed during the current study are available from the corresponding author on reasonable request.

## Abbreviations

BLSA: The Beijing Longitudinal Study of Aging
SBP: Systolic Blood Pressure
DBP: Diastolic Blood Pressure
PP: Pulse Pressure
BMI: Body Mass Index
TC: Total Cholesterol
TG: Triglyceride
LDL-C: Low Density Lipoprotein Cholesterol
HDL-C: High Density Lipoprotein Cholesterol
HCY: Homocysteine
UA: Uric Acid
PCR: Polymerase Chain Reaction
DNA: Deoxyribonucleic Acid
MI: Myocardial Infarction
CI: Confidence Interval
AD: Alzheimer Disease
ADL: Activities of Daily Living
IADL: Instrumental Activities Daily Living
GDS: The Geriatric Depression Scale
MMSE: Mini-Mental State Examination
ApoE: Apolipoprotein E
GLM: General Line Model
