# Supplementary material for: Combined homocysteine and apoE rs429358 and rs7412 polymorphism in association with serum lipid levels and cognition in Chinese community-dwelling older adults
Source: BMC Psychiatry. 2022 Mar 29;22:223. doi: 10.1186/s12888-022-03877-4 (PMC8966244; doi:10.1186/s12888-022-03877-4)
Supplement: Supplementary file 1 — Additional file 1. [file 12888_2022_3877_MOESM1_ESM.docx]

**Supplementary Table 1.** Subject serum parameters according to ApoE genotype in Chinese adults.

|  | ApoE rs429358(C/T) | ApoE 429358(T/T) | *p* value | ApoE rs7412(C/C) | ApoE rs7412(C/T)) | *p* value |
| --- | --- | --- | --- | --- | --- | --- |
| N | 184 | 1043 |  | 1020 | 214 |  |
| Fasting blood sugar, mmol/L | 5.62(5.36,5.88) | 5.69(5.57,5.80) | 0.625 | 5.69(5.57,5.81) | 5.62(5.38,5.87) | 0.596 |
| TC, mmol/L | 5.78(5.61.5.96) | 5.83(5.76,5.91) | 0.576 | 5.56(5.40.5.72) | 5.87(5.67,5.95) | <0.000* |
| TG, mmol/L | 1.59(1.42,1.76) | 1.55(1.48,1.63) | 0.719 | 1.53(1.45,1.61) | 1.72(1.56,1.88) | 0.031* |
| LDL-C, mmol/L | 2.96(2.85,3.08) | 2.92(2.87,2.97) | 0.458 | 2.99(2.94,3.04) | 2.59(2.48,2.69) | <0.000* |
| HDL-C, mmol/L | 1.20(1.16,1.24) | 1.23(1.21,1.25) | 0.179 | 1.22(1.20,1.24) | 1.25(1.21,1.29) | 0.249 |
| HCY, mmol/L | 22.65(19.76,25.55) | 22.61(21.34,23.88) | 0.976 | 22.37(21.06,23.67) | 24.42(21.75,27.09) | 0.158 |

Data were expressed as mean (95% CI). The General Line Model (GLM) univariate analysis was used for data analysis. Factors including sex, age, BMI, smoking, alcohol drinking and physical activity were adjusted. TC: Total Cholesterol; TG: Triglyceride; HDL-C: High-density Lipoprotein Cholesterol; LDL-C: Low-density Lipoprotein Cholesterol; HCY: Homocysteine; UA: Uric Acid; **p*< 0.05 was considered as significance.

**Supplementary Table 2. Cognition according to ApoE genotype in Chinese adults**

|  | ApoE rs429358(C/T) | ApoE rs429358(T/T) | *p* value | ApoE rs7412(C/C) | ApoE rs7412(C/T)) | *p* value |
| --- | --- | --- | --- | --- | --- | --- |
| N | 184 | 1043 |  | 1020 | 214 |  |
| Orientation | 8.40(8.08,8.72) | 8.68(8.54,8.88) | 0.100 | 8.71(8.56,8.85) | 8.33(8.04,8.62) | 0.018* |
| Memory and delayed recall | 5.11(4.93,5.29) | 5.30(5.22,5.37) | 0.053 | 5.27(5.19,5.38) | 5.31(5.15,5.52) | 0.473 |
| Attention | 3.88(3.61.4.14) | 3.91(3.80.4.03) | 0.796 | 3.92(3.81.4.05) | 3.79(3.55.4.04) | 0.303 |
| Language | 6.36(6.17,6.55) | 6.50(6.42,6.58) | 0.164 | 6.52(6.43,6.60) | 6.54(6.36,6.71) | 0.849 |
| Visual and executive | 0.62(0.55,0.69) | 0.65(0.63,0.68) | 0.311 | 0.66(0.63,0.69) | 0.61(0.55,0.67) | 0.104 |
| MMSE score | 24.36(23.58,25.14) | 25.04(24.70,25.38) | 0.102 | 25.05(24.70,25.40) | 24.52(23.81,25.23) | 0.166 |

Note:MMSE: Mini-Mental State Examination; ApoE: Apolipoprotein E. **p*< 0.05 was considered as significance. The General Line Model (GLM) univariate

analysis was used for data analysis. Factors including sex, age, BMI, education, smoking, alcohol drinking and physical activity were adjusted.

**Supplementary Table 3. Dietary intakes according to tHcy in Chinese adults**

|  | Total | HCY≤15 | HCY>15 | X^2^ | *p* value |
| --- | --- | --- | --- | --- | --- |
| N | 1458 | 556 | 902 |  |  |
| Red meat (≥once a week ) | 795 (54.5) | 323 (58.1) | 472 (52.3) | 5.56 | 0.018 |
| Chicken/Duck meat (≥once a week ) | 455 (31.2) | 197 (35.4) | 258 (28.6) | 11.05 | 0.004 |
| Fish/shrimp (≥twice a month) | 304 (20.9) | 142 (25.5) | 162 (18.0) | 13.26 | 0.001 |
| Egg (≥one egg/per day) | 784 (53.8) | 332 (59.7) | 452 (50.1) | 20.28 | 0.000 |
| Milk (≥once per day) | 342 (23.5) | 163 (29.3) | 179 (19.8) | 24.73 | 0.000 |
| Bean products (≥once a week ) | 742 (50.9) | 307 (55.2) | 435 (48.2) | 6.83 | 0.033 |
| Vegetables (≥once per day) | 1422 (97.5) | 542 (97.5) | 880 (97.6) | 2.66 | 0.265 |
| Fruits (≥once per day) | 633 (43.4) | 272 (48.9) | 361 (40.0) | 13.53 | 0.001 |
| Tea (≥once per day) | 527 (36.1) | 196 (35.3) | 331 (36.7) | 1.76 | 0.414 |
| Staple food (≥300 g/per day) | 664 (45.5) | 224 (40.3) | 440 (48.8) | 12.57 | 0.002 |
| Taste (salty taste preference) | 541 (37.1) | 176 (31.7) | 365 (40.5) | 11.51 | 0.003 |

**p*< 0.05 was considered as significance.
